# Absolutist word usage in spoken language as a marker of depression: an ecological momentary assessment study

**DOI:** 10.1186/s12888-026-08575-z

**Published:** 2026-08-31

**Authors:** Jonathan F. Bauer, Maurice Gerczuk, Lena Schindler-Gmelch, Björn Schuller, Matthias Berking

**Affiliations:** 1 Department of Clinical Psychology and Psychotherapy, Friedrich-Alexander- Universität Erlangen-Nürnberg, 91052 Erlangen, Germany; 2 CHI – Chair of Health Informatics, Technical University of Munich, 81675 Munich, Germany; 3 GLAM - Group on Language, Audio, & Music, Imperial College London, London, SW7 2AZ UK

**Keywords:** Depression, Absolutist words, Speech, Cognitive distortions, Ecological momentary assessment, Monitoring, Emotion regulation, Linguistics

## Abstract

**Background:**

Cognitive distortions are central to the maintenance of depression, as they bias information processing and negatively impact adaptive emotion regulation. As one manifestation of cognitive distortions, usage of absolutist words (e.g., always, never, must, completely) in written texts has been found to be indicative of underlying depression. Since absolutist word usage may allow important insights into maladaptive thinking patterns, it could be a relevant target for both monitoring and treating depressive symptoms. Therefore, we tested the relationships between absolutist word usage in spoken language with depression diagnosis, depressive symptom severity and current depressed mood.

**Method:**

We recruited 144 age- and gender-matched participants with clinical depression (*n* = 48), subclinical depression (*n* = 48), and no history of depression (*n* = 48). By conducting smartphone-based ecological momentary assessments (EMA) three times daily for two weeks, participants provided ratings of depressed mood and speech samples, which included mood descriptions and mood-regulating statements. The Hamilton Rating Scale for Depression (HRSD) was administered at the end of the 2-week period to assess depressive symptom severity retrospectively. Group differences were calculated with ANOVAs, associations between depressed mood and absolutist word usage were evaluated with multilevel models, and the relationship between depressive symptom severity and absolutist word usage was calculated with regression models.

**Results:**

Results showed more frequent absolutist word usage over the EMA phase in individuals with clinical depression compared to individuals with no history of depression. Furthermore, absolutist word usage was *negatively* associated with momentary depressed mood among individuals with elevated depressive symptoms. Additional analyses testing the temporal relationship showed that greater absolutist word usage was associated with higher levels of depressed mood after 12–24 h. Accordingly, absolutist word usage was positively associated with depressive symptom severity assessed after the 2-week period.

**Conclusion:**

Our results have several implications: First, absolutist word usage in spoken language appears to be an indicator of depression, which may be relevant for the optimization of depression assessment and monitoring approaches. Second, the finding that absolutist word usage was associated with *lower* depressed mood in the short-term, but *higher* depressed mood in the long-term provides important insights into the mechanisms of depression, and may help identify relevant treatment targets for clinicians.

**Trial registration:**

German Clinical Trial Registration DRKS00023670 on 19/01/2021 (https://drks.de/search/en/trial/DRKS00023670).

**Supplementary Information:**

The online version contains supplementary material available at https://doi.org/10.1186/s12888-026-08575-z.

## Introduction

Major depressive disorder (MDD), a leading cause of disability worldwide [1], is defined by symptoms of depressed mood, loss of pleasure and/or interest, and behavioral, physiological, as well as cognitive alterations such as reduced activity, disturbed sleep, and suicidal ideation [2]. It has shown that MDD can be treated effectively with Cognitive Behavioral Therapy (CBT) [3, 4]. However, while many patients benefit from these treatments, studies have found substantial rates of insufficient treatment response, non-response, and disease recurrence in others [5–8]. Gaining a deeper understanding of MDD to better assess and monitor key underlying mechanisms of the disorder may help to personalize and further optimize treatment [9].

CBT for depression is substantially influenced by Beck’s cognitive model, which proposes that the occurrence of cognitive distortions in MDD impact the view of the self, the world, and the anticipated future [10]. Cognitive distortions in MDD represent depressotypic thinking styles associated with cognitive inflexibility and an overemphasis on unpleasant information. This is often characterized by extreme dichotomies or overgeneralizations that ultimately result in rigid and dysfunctional beliefs [11]. Empirically, cognitive distortions in MDD have been shown to lead to an overly strong focus on negative information and an inability to inhibit negative information, resulting in a perseverance of depressive symptoms [12]. Thus, a main goal in MDD-focused CBT is to identify, address, and change cognitive distortions, dysfunctional beliefs, and ultimately depressogenic schemas to achieve a lasting reduction in depressive symptomatology [13]. Moreover, evidence suggests that cognitive distortions and emotional dysregulation might not only be problematic for those with acute MDD. Biased cognitive processing has also been observed in individuals with remitted MDD, individuals with subclinical symptoms, and even in individuals with experimentally induced depressed mood [14]. Furthermore, in patients with remitted MDD, an absolutist, dichotomous thinking style increased the probability of a depressive relapse [15]. In sum, cognitive distortions appear to affect patients with current MDD, recovered patients, and individuals at risk of developing MDD. Thus, it would be beneficial to develop valid and reliable methods to accurately detect and monitor cognitive distortions.

Empirically, depression has been associated with altered speech patterns [16]. In particular, absolutist or dichotomous thinking styles may be reflected in the use of language that conveys totality or certainty, with little or no nuance. In a recent study, a team of linguists and psychologists developed a list of absolutist words (e.g., always, never, must, completely) that express totality in magnitude or probability, rather than allowing for probabilistic or gradual description. They then searched for these words in online forums. Their research results showed that absolutist words were in fact used more frequently in depression forums compared to control forums [17]. Another study found that absolutist word frequency in online searches was associated with population-wide increases in depression scores during the COVID-19 pandemic [18]. These results support the assumption that absolutist word usage reflects dichotomous, rigid beliefs that are involved in depressotypic cognitive distortions. While measuring absolutist word usage promises direct insights into mechanisms of MDD, previous studies have only indirectly measured the association between absolutist word usage and depressive symptoms. To the best of our knowledge, no research to date has been conducted to study absolutist word usage in spoken language and its relation to MDD. Therefore, in the current study, we test whether individuals with current MDD use absolutist words more frequently compared to non-depressed individuals. Furthermore, we test the predictive value of absolutist word usage in spoken language on depressed mood and depressive symptom severity. In addition, absolutist word use will be analyzed in relation to the valence of speech content to assess potential effects of sentiment. Since cognitive distortions have been observed not only in individuals with current MDD, but also in those at risk of developing MDD, we examine said associations in individuals meeting criteria for MDD, individuals with subclinical depression, and individuals with no history of depression. We expect that individuals meeting criteria for MDD more frequently use absolutist words. We further hypothesize that a higher frequency of absolutist word usage predicts elevated depressed mood and increased depressive symptom severity.

## Method

### Participants and procedures

For initial screening, participants’ depressive symptom severity was assessed with the depression module of the Patient Health Questionnaire (PHQ-9; [19]). We recruited 144 participants in total, with 48 meeting criteria for MDD, 48 reporting elevated, yet subclinical (SC) depressive symptoms (PHQ-9 ≥ 5), and 48 never-depressed (ND) participants (PHQ-9 < 5, no self-reported prior history of MDD). Current diagnoses and lifetime history of MDD were assessed using the Structured Clinical Interview for DSM-5 (SCID-5-CV, [20]). We matched the groups for gender, age, and education. Participants were eligible for inclusion if they were at least 18 years old and provided informed consent. Participants were excluded if they had a current diagnosis of bipolar, psychotic, or substance-related disorder (except for nicotine) within the past six months as these disorders may significantly impair the ability to participate in or adhere to the study procedures. Otherwise, exclusion criteria were kept to a minimum to maximize the external validity of the study. Additionally, participants were excluded if they had received any psychotherapeutic treatment during the past six months, as required for another component of the study. Depending on the total number of completed assessments, participants received up to 150€ for their participation. Participants from the MDD sample were offered CBT at the affiliated outpatient clinic for psychotherapy subsequent to study participation. Participants’ mean age was 32.72 years (ranging from 20 to 63 years, *SD* = 11.02). The majority of participants (67%) were female. An overview of sociodemographic and comorbidity data is shown in Table 1. The data reported here are part of a larger study investigating speech patterns associated with MDD in order to improve disease assessment and treatment [21, 22]. Further details regarding the sample size calculation and study flow are provided in [22]. Participants were recruited from the waiting list of the university’s outpatient psychotherapeutic clinic by advertising on the official website, posting on social media platforms, and circulating flyers among local psychiatrists as well as directly to prospective participants in public spaces. An initial online screening questionnaire was completed by potential participants, followed by an in-person diagnostic Session (t0), during which final eligibility was assessed (see assessment section for details). If participants were included, an assessment Session (t1) was conducted that included administration of the Hamilton Rating Scale for Depression (HRSD [23]) to determine baseline severity of depressive symptoms. In addition, participants provided several speech samples on site (see [22]). Since these speech samples were not used for the present study, we do not report on them in detail. At the end of Session t1, participants received a study smartphone (Motorola G8 Lite) with an app specifically developed by our research team to record speech via ecological momentary assessments (EMA). EMAs were prompted three times a day, randomly within three time intervals between 8 am and 10 am, between 12 pm and 2 pm, and between 4 pm and 6 pm. For each assessment, participants first received the three questions regarding their current mood state and potential regulation efforts: Question 1: “Wie geht es Ihnen im Moment?” (translation: “How are you feeling at the moment?”), Question 2: “Wie kommen Sie mit diesem Gefühl klar?” (translation: “How are you coping with this feeling?”), Question 3: “Wie wollen Sie mit diesem Gefühl umgehen?” (translation: “How would you like to cope with this feeling?”). These questions were followed by an assessment of depressed mood (“Bitte geben Sie an, wie ausgeprägt Ihre depressive Stimmung im Moment ist”; translation: “Please indicate how severe your depressive mood is at the moment”) using an 11-point visual analogue scale. Finally, participants received the instruction to share a thought that would improve their current mood, and to repeat this positive thought two more times. Participants were instructed to make audio recordings of themselves whilst reading and responding to each of the three questions and whilst uttering their positive thoughts. This procedure of assessing mood states and regulation efforts was designed specifically for this study. It was aimed at gaining insight into central aspects of participants’ mood regulation efforts (i.e., awareness, acceptance, readiness to confront, and modification [24]) based on the Adaptive Coping with Emotions model [25]. A detailed overview of the EMA procedure is shown in Figure S1 of the Supplemental Material. After two weeks of EMA, participants returned to an additional Session (t2) that included another HRSD assessment to determine depressive symptom severity. At this session, the EMA phase concluded, and participants returned the study smartphones to the research team. All study procedures were conducted in accordance with the Declaration of Helsinki and approved by the ethics committee of the Friedrich-Alexander-Universität Erlangen-Nürnberg. The trial was pre-registered in the German clinical trials registry under https://drks.de/search/en/trial/DRKS00023670 during data collection and prior to data analyses. Hypotheses for this study were registered after data collection under https://osf.io/8uxh4.

**Table 1:** Sociodemographic data, depressive symptom severity, and comorbid disorders

	MDD sample	SC sample	ND sample
Age	32.98 (11.10)	32.22 (10.63)	32.85 (11.55)
Sex, female, *n* (%)	32 (66.7)	32 (66.7)	32 (66.7)
Highest education, *n* (%)			
* No academic degree*	1 (2.1)	--	--
* High school degree*	20 (41.7)	24 (50.0)	24 (50.0)
* University degree*	27 (56.3)	24 (50.0)	24 (50.0)
HRSD pre-EMA (Mean, SD)	14.83 (6.45)	4.83 (3.69)	1.23 (1.60)
HRSD post-EMA (Mean, SD)	15.29 (5.81)	4.33 (3.93)	1.44 (1.75)
Previous MDE, *n* (%)	18 (37.5)	12 (25.0)	--
Current comorbid disorders	26 (54.2)	2 (4.2)	--
* Anxiety disorders*	18 (37.5)	--	--
*Obsessive-compulsive* * disorder*	8 (16.7)	--	--
* Attention deficit* * hyperactivity disorder*	6 (12.5)	--	--
* Eating disorder*	--	1 (2.1)	--
* Adjustment disorder*	--	1 (2.1)	--

Note. MDD sample = participants meeting criteria for major depressive disorder; SC sample = participants with subclinical depressive symptoms; ND sample = non-depressed participants; HRSD = Hamilton Rating Scale for Depression; MDE = major depressive episode. EMA = ecological momentary assessment

### Assessments

During the diagnostic Session t0, the German version of the Structured Clinical Interview for DSM-5 ([20], German version: [26]) to assess diagnostic status of participants. Trained interviewers administered the SCID-5-CV under the supervision of a senior clinical diagnostician/therapist. A blinded and experienced rater second-rated 10% of the videotaped diagnostic interviews to assess interrater-reliability. Excellent agreement regarding the presence or absence of an MDD diagnosis was indicated by a Cohen’s κ of 0.84.

In addition, depressive symptom severity was assessed with a semi-structured, 17-item version of the HRSD [27], conducted at t1 and t2. The HRSD is a commonly used clinical interview, consisting of items rated on a scale of 0–4 or 0–2, depending on the item (total score ranging from 0 to 52) [23]. The HRSD allows reliable and valid assessment of depressive symptom severity, specifically if it is conducted as a structured interview [28]. A blinded and experienced rater second-rated 10% of the videotaped interviews. Pearson’s correlation coefficient for mean scores was 0.99, which indicates excellent interrater reliability.

### Absolutist word usage

Absolutist words are defined as terms that express totality in probability or magnitude, allowing little or no gradation or nuance. The list of English absolutist words developed and validated by Al-Mosaiwi & Johnstone [17] was translated into German by two native German speakers with high proficiency in English and subsequently back-translated by a third translator. The original and back-translated versions showed 95% agreement. The final list of German absolutist words, shown in Table S1 of the Supplemental Material, included all relevant conjugated and declined forms of each word. All EMA recordings, including participants’ reports of their current mood state, coping efforts, and positive thoughts, were automatically transcribed with ‘Whisper’ [29], an automated speech recognition system. The output was saved to a csv-file and absolutist words were counted with Microsoft Excel. For each assessment, absolutist word usage was calculated by dividing the amount of absolutist words by the total amount of words, leading to a score between 0 (no absolutist word was used) and 1 (only absolutist words were used). The applied formulas are described in Table S2 of the Supplemental Material.

### Sentiment analysis

To analyze absolutist word usage in the context of valence, a sentiment analysis was conducted with a specialized German BERT-based model [30] that allowed a categorization of transcripts from speech recordings into the categories ‘positive’, ‘negative’, and ‘neutral’. This model is pre-trained on over 1.8 million German social media reviews and comments.

### Statistical analyses

To ensure that automated transcriptions allowed for valid calculation of absolutist word usage, absolutist words were manually counted in a random subsample of *n* = 50 speech recordings. Then, agreement between manually and automatically derived absolutist word counts were calculated with a Pearson Correlation. Subsequently, we tested hypotheses in three parts: First, we tested differences in overall absolutist word usage and frequency of sentiment categories between samples. Second, we tested whether absolutist word usage and its interaction with valence predicted depressed mood for individual assessments nested within individuals. Third, we tested the relationship between absolutist word usage averaged across all assessments and depressive symptom severity. For the first part, absolutist word usage was compiled across all speech recordings for each participant. Then, differences were tested in overall absolutist word usage between samples using an ANOVA. Furthermore, frequencies of valence categories were aggregated across all speech recordings for each participant and differences between samples were examined using ANOVAs. For the category ‘negative’, a Welch ANOVA was conducted because the assumption of homoscedasticity was violated. For the second part, the percentage of absolutist word usage was calculated across speech recorded at each individual EMA time point. We calculated hierarchical multilevel models to test the predictive value of absolutist word usage and valence for depressed mood nested within individuals. Absolutist word usage and valence was determined for each individual EMA time point and matched with the concurrent depressed mood rating. We analyzed all available cases, deleting missing time points. We tested and compared a model including only control variables (sex, age, presence of comorbid disorders), a model that additionally included main effects, and a model that added interactions. We included person means of depressed mood to account for between-person effects and person-mean-centered depressed mood to account for within-person effects. For the third part, we computed a hierarchical regression model to test the association between absolutist word usage, averaged across assessments, and depressive symptom severity assessed with the HRSD after the EMA phase. We first included the control variables mentioned above, then added absolutist word usage, and added baseline HRSD in the final step.

Statistical analyses were conducted with RStudio using the “lme4” package [31] and the “lmeTest” package [32] for the regression models. We used two-sided tests with a significance level of 5% for all analyses.

## Results

Sociodemographic data for the three samples are shown in Table 1. The total counts of absolutist words in the speech recordings for each sample are presented in Table S1 of the Supplemental Material.

### Validation of automated transcriptions

To ensure that automated transcriptions allowed for valid calculation of absolutist word usage, absolutist words were manually counted in a random subsample of *n* = 50 speech recordings. Agreement between manually and automatically derived absolutist word counts was very high (*r* = .93).

### Comparison of absolutist word usage and valence between samples

An ANOVA revealed a significant difference in absolutist word usage between samples (*F*(2,141) = 4.51, *p* = .013). A post-hoc analysis showed that the MDD sample (*M* = 2.12%, *SD* = 0.98%) used absolutist words significantly more frequently compared to the ND sample (*M* = 1.60%, *SD* = 0.79%, *p* = .015). The SC sample (*M* = 2.03%, *SD* = 0.90%) showed no significant differences in absolutist word usage compared to both the MDD sample (*p* = .869) and the ND sample (*p* = .057).

Additional ANOVAs revealed significant differences between samples in the frequency of speech recordings with both negative (*F*(2,83.81) = 60.57, *p* < .001) and positive (*F*(2,141) = 32.54, *p* < .001) valence. Post hoc tests indicated that recordings from the MDD sample (negative: *M* = 19.71, *SD* = 8.69; positive: *M* = 16.87, *SD* = 9.04) were significantly more likely to have negative valence and less likely to have positive valence than recordings from both the SC sample (negative: *M* = 11.33, *SD* = 6.78, *p* < .001; positive: *M* = 25.35, *SD* = 8.74, *p* < .001) and the ND sample (negative: *M* = 5.27, *SD* = 3.85, *p* < .001; positive: *M* = 30.67, *SD* = 7.49, *p* < .001). Recordings from the SC sample were also significantly more likely to have negative valence and less likely to have positive valence than recordings from the ND sample (negative: *p* < .001; positive: *p* = .007). No significant differences were observed between samples in the frequency of recordings with neutral valence (*F*(2,141) = 2.87, *p* = .085).

**Table 2:** Results of multilevel models predicting ratings of depressed mood

Predictor	Model 0	Model 1 (+ main effects)	Model 2 (+ interactions)
	B (SD)	B (SD)	B (SD)
Step 1			
Age	0.01 (0.01)	0.01 (0.01)	0.01 (0.01)
Sex	-0.14 (0.33)	0.04 (0.31)	0.04 (0.31)
Anxiety disorder	1.88 (0.55)***	1.36 (0.50)**	1.39 (0.50)**
ADHD	0.68 (0.86)	0.64 (0.77)	0.65 (0.78)
OCD	0.80 (0.82)	1.12 (0.74)	1.09 (0.75)
Step 2			
Absolutist word usage - B		30.91 (14.79)*	31.48 (14.86)*
Absolutist word usage - W		-1.73 (0.94)	0.58 (3.16)
Sentiment (reference: Neutral)			
Negative		0.47 (0.06)***	0.46 (0.06)***
Positive		-0.29 (0.06)***	-0.30 (0.06)***
Step 3			
Simple slopes (absolutist word usage)			
when Negative			-4.38 (1.41)**
when Positive			-0.66 (1.06)
when Neutral			0.59 (3.15)
Model comparisons (*χ²*)		535.71***	6.33*

*Notes.* Random Slopes for absolutist word usage in all models. ADHD = attention deficit hyperactivity disorder; OCD = obsessive-compulsive disorder; B = Between-person-effect; W = Within-person-effect, * *p* < .05. ** *p* < .01. *** *p* < .001

### Relationship between absolutist word usage and depressed mood

Participants provided speech recordings at an average of 39.3 (*SD* = 9.2) EMA time points. The multilevel models testing whether absolutist word usage, valence, and their interaction predicted depressed mood are shown in Table 2. Results revealed that including interactions between absolutist word usage and valence significantly improved the model fit compared to only including main effects (*χ²* = 6.33, *p* = .042). Testing the interaction effects included in Model 3 more specifically with simple slopes analyses showed that absolutist word usage negatively predicted depressed mood within individuals if a recording had a negative valence (estimate = -4.38, *SD* = 1.41, *z* = − 3.11, *p* = .002). This means that in recordings with negative valence, greater absolutist word usage predicted lower depressed mood. In addition, results showed a significant between-person effect of absolutist word usage on depressed mood (estimate = 31.48, *SD* = 14.86, *t*(137) = 2.11, *p* = .036), suggesting that individuals with greater absolutist word usage reported greater depressed mood. The effects of the usage of individual absolutist words in the context of valence, tested with multilevel models, are shown in Table S3 of the Supplemental Material.

As the finding about a negative effect of absolutist word usage on depressed mood in recordings with negative valence was contrary to our hypothesis, we conducted additional analyses to examine the temporal relationship between absolutist word usage and depressed mood. Table 3 shows the lagged effects of absolutist word usage and valence on depressed mood at t0 + 1, t0 + 2, and t0 + 3. Model comparisons revealed that including the interaction between absolutist word usage and valence did not improve model fit relative to models including only the main effects. Therefore, only the main effects are reported in Table 3. Furthermore, when predicting depressed mood at t0 + 1, neither absolutist word usage at t0 nor valence at t0 explained additional variance beyond depressed mood at t0. However, when predicting depressed mood at t0 + 2, results revealed a positive main effect of absolutist word usage at t0 (estimate = 2.22, *SD* = 0.70, *t*(5232) = 3.25, *p* = .001) and positive valence at t0 (estimate = 0.12, *SD* = 0.06, *t*(5239) = 2.00, *p* = .046) in addition to the effect of depressed mood at t0 (estimate = 0.24, *SD* = 0.01, *t*(5234) = 17.22, *p* < .001), indicating that greater use of absolutist words and positive valence predicted higher levels of depressed mood two observations later. Similarly, when predicting depressed mood at t0 + 3, a significant positive main effect of absolutist word usage at t0 emerged (estimate = 1.39, *SD* = 0.70, *t*(5088) = 1.98, *p* = .048) additionally to the effect of depressed mood at t0 (estimate = 0.14, *SD* = 0.01, *t*(5090) = 9.85, *p* < .001).

Taken together, the significant interaction between absolutist word usage and negative valence, shown in Table 2, showed a negative effect of the interaction between absolutist word usage and negatively valence and depressed mood at the same EMA time point. In contrast, the significant main effects reported in Table 3 indicate that greater absolutist word usage predicted higher levels of depressed mood two and three EMA time points later (t0 + 2 and t0 + 3). These findings suggest that, although greater absolutist word usage in negative utterances is associated with lower concurrent depressed mood, greater absolutist word usage predicts increases in depressed mood approximately 12–24 h later.

**Table 3:** Results of multilevel models to predict ratings of depressed mood at t0 + 1, t0 + 2, and t0 + 3

Predictor	Lag 1 (t0 + 1)	Lag 2 (t0 + 2)	Lag 3 (t0 + 3)
	B (SD)	B (SD)	B (SD)
Depressed mood t0	0.44 (0.01)***	0.24 (0.01)***	0.14 (0.01)***
Absolutist word usage – W	0.21 (0.63)	2.22 (0.68)**	1.39 (0.70)*
Sentiment (reference: Neutral)			
Negative	-0.10 (0.06)	0.03 (0.06)	< 0.01 (0.06)
Positive	0.04 (0.06)	0.12 (0.06)*	0.05 (0.06)

*Notes.* W = Within-person-effect, * *p* < .05. ** *p* < .01. *** *p* < .001

To examine the temporal direction of this association, additional analyses tested the lagged effect of depressed mood on subsequent absolutist word usage. Results showed that depressed mood at t0 (estimate = < 0.01, *SD* = < 0.01, *t*(5410) = -1.07, *p* = .286) did not explain additional variance in absolutist word usage at t0 + 1 beyond absolutist word usage at t0 (estimate = 0.04, *SD* = 0.01, *t*(5381) = 3.07, *p* = .002). Likewise, depressed mood at t0 did not significantly predict absolutist word usage at t0 + 2 (estimate = < 0.01, *SD* = < 0.01, *t*(5268) = -0.08, *p* = .934) or t0 + 3 (estimate = < 0.01, *SD* = < 0.01, *t*(5098) = -0.14, *p* = .892). These findings suggest a unidirectional temporal relationship in which absolutist word usage predicts subsequent depressed mood, whereas depressed mood does not predict later absolutist word usage.

**Table 4:** Results of multiple regression models predicting depressive symptom severity post EMA

Predictor	Model 0	Model 1	Model 2
	B (SD)	B (SD)	B (SD)
Step 1			
Age	0.58 (1.15)	1.03 (1.15)	-0.21 (0.70)
Sex	0.05 (0.05)	0.06 (0.05)	-0.01 (0.03)
Anxiety disorder	7.72 (1.88)***	7.2 (1.86)***	-0.70 (1.24)
ADHD	2.49 (2.95)	2.81 (2.91)	-0.33 (1.77)
OCD	6.04 (2.84)*	6.36 (2.80)*	5.03 (1.70)**
Step 2			
Absolutist word usage		137.20 (58.79)*	21.48 (36.37)
Step 3			
Baseline depressive symptom severity			0.82 (0.05)***

*Notes.* ADHD = attention deficit hyperactivity disorder; OCD = obsessive-compulsive disorder; * *p* < .05. ** *p* < .01. *** *p* < .001

### Associations with depressive symptom severity

Regression analyses shown in Table 4 revealed that absolutist word usage was a significant predictor of depressive symptom severity assessed with the HRSD at the end of the EMA phase additionally to sociodemographic variables and comorbid disorders (estimate = 137.20, *SD* = 58.79, *p* = .021). When including depressive symptom severity assessed with the HRSD prior to the EMA phase, no significant effect of absolutist word usage was found (estimate = 21.48, *SD* = 36.37, *p* = .556), suggesting that absolutist word usage during the EMA phase did not predict a change in depressive symptom severity. It should be noted that depressive symptom severity at the beginning of the EMA phase was largely overlapping and therefore highly predictive of depressive symptom severity after the EMA phase (estimate = 0.82, *SD* = 0.05, *p* < .001).

## Discussion

Absolutist word usage has been found to be indicative of depression in written text [17, 18], reflecting cognitive distortions, such as overgeneralizing and dichotomous thinking, as central mediators in the development and maintenance of depressive symptoms [10]. The current study showed that absolutist word usage indicates depression and depressed mood in spoken language during emotion recognition and regulation efforts. The current study investigated whether absolutist word usage in the context of emotional valence indicates depression and depressed mood during emotion recognition and regulation efforts in spoken language. Consistent with our hypotheses, absolutist word usage over a two-week EMA phase differed between individuals with clinical depression and individuals with no history of depression and was associated with depressive symptom severity assessed at the end of the EMA phase. Surprisingly, when an utterance was negatively valenced, more frequent absolutist word usage was associated with lower momentary depressed mood within individuals. Further analyses of the temporal relationship showed that this association turns after 12–24 h, making absolutist word usage a positive predictor of depressed mood in the longer term.

Our results support theoretical assumptions about cognitive distortions in MDD by showing that individuals with depression use absolutist words more frequently compared to non-depressed individuals during a two-week EMA phase. In parallel, greater use of absolutist words predicted higher depressive symptom severity assessed at the end of the EMA phase. Contrary to our expectations, we found that *within* individuals, greater use of absolutist words in a negatively valenced utterance was associated with lower momentary depressed mood, but greater use of absolutist word usage also predicted an increase in depressed mood in ratings occurring around 12–24 h later. We propose that absolutist word usage may function as a maladaptive emotion regulation strategy, which typically downregulate unpleasant emotions in the short-term, but lead to the maintenance of psychopathology in the long-term [33]. Absolutist words may represent overgeneralizations or exaggerations due to a lack of emotional differentiation or because they enable individuals to avoid unpleasant internal experiences, both of which are associated with depressive symptoms [34, 35]. This explanation aligns with previous research describing overgeneral autobiographical memory bias [36] as a strategy to avoid negative affective memories, which leads to elevated rumination and, thus, maintains depressive symptomatology [37]. Our results suggest that this depressotypic bias in overgeneral information processing may also relate to recent or current emotional experiences, as reflected in the more frequent use of absolutist words. Analyses of individual absolutist words suggest that the pattern of a short-term negative association coupled with a longer-term positive association with depressed mood was present only in specific absolutist words that may be categorized specifically as overgeneralizations (e.g., *ganz*, translation: *whole).* Other absolutist words that had a significant effect on depressed mood had a positive effect on depressed mood in both the long and the short term may be categorized as exaggerations (e.g., absolut, translation: absolutely).

Our study has several important implications for clinical practice. While identifying and changing cognitive distortions is common practice in CBT, and particularly so in the context of MDD, our study enabled direct observations and objective measuring of such distortions in the form of absolutist word usage in spoken language. Our approach may facilitate the assessment and monitoring of depressive mood, inform therapists and patients about the use of absolutist words by illustrating the frequency, consistency, and impact of absolutist word usage, and allow online feedback of absolutist word usage for digital health applications such as just-in-time adaptive interventions [38].

Limitations of our study are the following: First, the translation of absolutist words may not fully preserve the semantic range of the original English terms. Most notably, the word *ganz* – the most frequently used word from the list of absolutist words, is a literal translation of *whole*, yet depending on context it can also function as a downtoner equivalent to *rather* or *quite*. In this latter usage, *ganz* expresses vagueness or approximation rather than categorical absolutism. This semantic ambiguity raises the question of whether its elevated frequency reflects genuine absolutist thinking or, alternatively, a tendency toward imprecise, overgeneralized language use. Importantly, however, this observation does not necessarily undermine the study’s findings. Rather, it suggests a conceptual refinement: the linguistic markers identified here may be better characterized as indicators of cognitive vagueness and overgeneralization than of superlatives or extreme exaggeration per se – a distinction that aligns with theoretical accounts of absolutist thinking as context-independent and unqualified by nuance [17]. Second, we made a very coarse-grained assessment of distorted cognitions by calculating the ratio of absolutist to total words used per assessment. Arguably, taking negations and complex semantic structures into account may lead to even better predictions of depressed mood and depressive symptom severity. However, we aimed for keeping the predictor variable straightforward and comprehensible, ensuring its interpretability to enhance the understanding and treatment of MDD. In contrast, this is typically not the case for natural language models that use machine learning to analyze lexical content [39]. Third, the automated transcriptions involved an unclear number of orthographic errors such as pseudohomophones resulting from dialectal or slurred speech that the software was unable to recognize accurately. However, a manual count of absolutist words in a random subsample showed very high agreement with the automated count. Furthermore, if any systematic bias between samples existed, we would expect more orthographic errors and, thus, less correctly identified absolutist words in the MDD sample, since previous data suggests that patients with MDD speak with less articulatory precision compared to non-depressed individuals [40]. In that case, our study would underestimate the ratio of absolutist word usage in MDD. However, the ongoing improvement of machine learning methods will improve automated transcription, mitigating this limitation in future research. Fourth, it should be noted that we did not directly assess the external validity of the proposed link between absolutist words and cognitive distortions. Future studies should therefore include measures of cognitive distortions to examine whether linguistic markers reflect underlying maladaptive cognitive processes and to provide additional validation for the proposed relationship. Lastly, results did not show incremental predictive value of absolutist word usage for depressive symptom severity assessed after the EMA phase when controlling for depressive symptom severity prior to the EMA phase. This may be attributed to little change in depressive symptomatology between the pre- and post-EMA assessments. Future research should test the predictive value of absolutist word usage on depressive symptom severity during episodes with expected change in psychopathology (e.g., during psychotherapy or pharmacotherapy).

In conclusion, our research demonstrates the significant potential of absolutist word usage as a marker of depressed mood and depressive symptom severity. Our results and the present approach to collect speech data may improve diagnostics and monitoring of depression and patients’ maladaptive emotion regulation strategies. Further, our results may inform targeted therapeutic interventions in response to patients’ absolutist utterances.

## Supplementary Information

Below is the link to the electronic supplementary material.


Supplementary Material 1


## Data Availability

The datasets analyzed during the current study can be made available from the corresponding author on reasonable request.

## Abbreviations

ADHD: Attention Deficit Hyperactivity Disorder
BERT: Bidirectional Encoder Repositories from Transformers
CBT: Cognitive Behavioral Therapy
EMA: Ecological Momentary Assessment
HRSD: Hamilton Rating Scale for Depression
MDE: Major Depressive Episode
MDD: Major Depressive Disorder
ND: Non-depressed
OCD: Obsessive-Compulsive Disorder
PHQ-9: depression module of the Patient Health Questionnaire
SC: Subclinically depressed
SCID-5-CV: Structured Clinical Interview for DSM-5 Disorder – Clinician Version

## References

[1] James SL, Abate D, Abate KH, Abay SM, Abbafati C, Abbasi N, Abbastabar H, Abd-Allah F, Abdela J, Abdelalim A, Abdollahpour I, Abdulkader RS, Abebe Z, Abera SF, Abil OZ, Abraha HN, Abu-Raddad LJ, Abu-Rmeileh NME, Accrombessi MMK, Murray CJL. Global, regional, and national incidence, prevalence, and years lived with disability for 354 diseases and injuries for 195 countries and territories, 1990–2017: a systematic analysis for the Global Burden of Disease Study 2017. Lancet. 2018;392(10159):1789–858. 10.1016/S0140-6736(18)32279-7. 30496104 PMC6227754

[2] American Psychiatric Association. Diagnostic and statistical manual of mental disorders: DSM-5. 5th ed. American Psychiatric Publishing; 2013. 10.1176/appi.books.9780890425596.

[3] Cuijpers P, Berking M, Andersson G, Quigley L, Kleiboer A, Dobson KS. A meta-analysis of cognitive-behavioural therapy for adult depression, alone and in comparison with other treatments. Can J Psychiatry. 2013;58(7):376–85. 10.1177/070674371305800702. 23870719

[4] Furukawa TA, Noma H, Caldwell DM, Honyashiki M, Shinohara K, Imai H, Churchill R. Waiting list may be a nocebo condition in psychotherapy trials: A contribution from network meta-analysis. Acta psychiatrica Scandinavica. 2014;130(3):181–92. 10.1111/acps.12275. 24697518

[5] Beshai S, Dobson KS, Bockting CLH, Quigley L. Relapse and recurrence prevention in depression: Current research and future prospects. Clin Psychol Rev. 2011;31(8):1349–60. 10.1016/j.cpr.2011.09.003. 22020371

[6] Casacalenda N, Perry JC, Looper K. Remission in major depressive disorder: A comparison of pharmacotherapy, psychotherapy, and control conditions. Am J Psychiatry. 2002;159(8):1354–60. 10.1176/appi.ajp.159.8.1354. 12153828

[7] Taylor DJ, Walters HM, Vittengl JR, Krebaum S, Jarrett RB. Which depressive symptoms remain after response to cognitive therapy of depression and predict relapse and recurrence? J Affect Disord. 2010;123(1–3):181–7. 10.1016/j.jad.2009.08.007. 19733912 PMC2860061

[8] Vittengl JR, Clark LA, Dunn TW, Jarrett RB. Reducing relapse and recurrence in unipolar depression: A comparative meta-analysis of cognitive-behavioral therapy’s effects. J Consult Clin Psychol. 2007;75(3):475–88. 10.1037/0022-006X.75.3.475. 17563164 PMC2630051

[9] Simon GE, Perlis RH. Personalized medicine for depression: Can we match patients with treatments? Am J Psychiatry. 2010;167(12):1445–55. 10.1176/appi.ajp.2010.09111680. 20843873 PMC3723328

[10] Beck AT, Bredemeier K. A Unified Model of Depression: Integrating clinical, cognitive, biological, and evolutionary perspectives. Clin Psychol Science: Journal Association Psychol Science. 2016;4(4):596–619. 10.1177/2167702616628523.

[11] Beck JS. Cognitive behavioural therapy: basics and beyond. 2nd ed. Guilford Press; 2011. 10.1017/S135246581200094X.

[12] Joormann J, Gotlib IH. Emotion regulation in depression: Relation to cognitive inhibition. Cognition Emot. 2010;24(2):281–98. 10.1080/02699930903407948. PMC2839199 20300538

[13] Clark DA. Cognitive restructuring. In: Hofmann SG, editor. The Wiley handbook of cognitive behavioral therapy. Wiley; 2014. pp. 1–22. 10.1002/9781118528563.wbcbt02.

[14] Peckham AD, McHugh RK, Otto MW. A meta-analysis of the magnitude of biased attention in depression. Depress Anxiety. 2010;27(12):1135–42. 10.1002/da.20755. 21049527

[15] Teasdale JD, Scott J, Moore RG, Hayhurst H, Pope M, Paykel ES. How does cognitive therapy prevent relapse in residual depression? Evidence from a controlled trial. J Consult Clin Psychol. 2001;69(3):347–57. 10.1037/0022-006X.69.3.347. 11495165

[16] Koops S, Brederoo SG, De Boer JN, Nadema FG, Voppel AE, Sommer IE. Speech as a biomarker for depression. CNS Neurol Disorders-Drug Targets. 2023;22(2):152–60. 10.2174/1871527320666211213125847. 34961469

[17] Al-Mosaiwi M, Johnstone T. In an Absolute State: Elevated Use of Absolutist Words Is a Marker Specific to Anxiety, Depression, and Suicidal Ideation. Clin Psychol Science: J Association Psychol Sci. 2018;6(4):529–42. 10.1177/2167702617747074. PMC6376956 30886766

[18] Adam-Troian J, Bonetto E, Arciszewski T. Using absolutist word frequency from online searches to measure population mental health dynamics. Sci Rep. 2022;12(1):2619. 10.1038/s41598-022-06392-4. 35173219 PMC8850545

[19] Kroenke K, Spitzer RL, Williams JB. Patient health questionnaire-9. 1999. 10.1037/t06165-000.

[20] First MB, Williams JBW, Karg RS, Spitzer RL. Structured clinical interview for DSM-5 disorders, clinician version (SCID-5-CV). American Psychiatric Association; 2016.

[21] Bauer JF, Gerczuk M, Schuller B, Berking M. Optimizing speech elicitation tasks for machine learning-based depression assessment. In T. Ahram & W. Karwowski, editors, Proceedings of the AHFE International Conference on Human Factors in Engineering and Human-Centered Computing. 2024;159. 10.54941/ahfe1005694.

[22] Bauer JF, Schindler-Gmelch L, Gerczuk M, Schuller B, Berking M. Prosody-Focused Feedback Enhances the Efficacy of Anti-Depressive Self-Statements in Depressed Individuals–A Randomized Controlled Trial. Behav Res Ther. 2025;184:104667. 10.1016/j.brat.2024.104667. 39700643

[23] Hamilton M. A rating scale for depression. J Neurol Neurosurg Psychiatry. 1960;23(1):56–62. 10.1136/jnnp.23.1.56. 14399272 PMC495331

[24] Grant M, Salsman NL, Berking M. The assessment of successful emotion regulation skills use: development and validation of an English version of the emotion regulation skills questionnaire. PLoS ONE. 2018;13(10):e0205095. 10.1371/journal.pone.0205095 PMC6169969 30281666

[25] Berking M, Whitley B. Affect regulation training: a practitioner’s manual. Springer; 2014. 10.1007/978-1-4939-1022-9.

[26] Beesdo-Baum K, Zaudig M, Wittchen HU. SCID-5-CV: Strukturiertes Klinisches Interview für DSM-5-Störungen - Klinische Version. Hogrefe. 2019.

[27] Miller IW, Bishop S, Norman WH, Maddever H. The modified hamilton rating scale for depression: reliability and validity. Psychiat Res. 1985;14(2):131–142. 10.1016/0165-1781(85)90057-5. 3857653

[28] Carrozzino D, Patierno C, Fava GA, Guidi J. The Hamilton Rating Scales for Depression: A Critical Review of Clinimetric Properties of Different Versions. Psychother Psychosom. 2020;89(3):133–50. 10.1159/000506879. 32289809

[29] Radford A, Kim JW, Xu T, Brockman G, Mcleavey C, Sutskever I. Robust speech recognition via large-scale weak supervision. ICML’23: Proceedings of the 40th International Conference on Machine Learning. 2023:28492–28518.

[30] Guhr O, Schumann AK, Bahrmann F, Böhme HJ. Training a broad-coverage German sentiment classification model for dialog systems. Proceedings of the Twelfth Language Resources and Evaluation Conference. 2020:1627–1632.

[31] Bates D, Mächler M, Bolker B, Walker S. Fitting linear mixed-effects models using lme4. J Stat Softw. 2015;67(1). 10.18637/jss.v067.i01.

[32] Kuznetsova A, Brockhoff PB, Christensen RHB. lmerTest package: tests in linear mixed effects models. J Stat Softw. 2017;82(13). 10.18637/jss.v082.i13.

[33] Aldao A, Nolen-Hoeksema S, Schweizer S. Emotion-regulation strategies across psychopathology: A meta-analytic review. Clin Psychol Rev. 2010;30(2):217–37. 10.1016/j.cpr.2009.11.004. 20015584

[34] Demiralp E, Thompson RJ, Mata J, Jaeggi SM, Buschkuehl M, Barrett LF, Ellsworth PC, Demiralp M, Hernandez-Garcia L, Deldin PJ, Gotlib IH, Jonides J. Feeling blue or turquoise? Emotional differentiation in major depressive disorder. Psychol Sci. 2012;23(11):1410–6. 10.1177/0956797612444903. 23070307 PMC4004625

[35] Spinhoven P, Drost J, de Rooij M, van Hemert AM, Penninx BW. A longitudinal study of experiential avoidance in emotional disorders. Behav Ther. 2014;45(6):840–50. 10.1016/j.beth.2014.07.001. 25311292

[36] Barnhofer T, de Jong-Meyer R, Kleinpass A, Nikesch S. Specificity of autobiographical memories in depression: An analysis of retrieval processes in a think-aloud task. Br J Clin Psychol. 2002;41(4):411–6. 10.1348/014466502760387524. 12437795

[37] Liu Y, Yu X, Yang B, Zhang F, Zou W, Na A, Zhao X, Yin G. Rumination mediates the relationship between overgeneral autobiographical memory and depression in patients with major depressive disorder. BMC Psychiatry. 2017;17(1):103. 10.1186/s12888-017-1264-8. 28327097 PMC5361774

[38] Nahum-Shani I, Smith SN, Spring BJ, Collins LM, Witkiewitz K, Tewari A, Murphy SA. Just-in-Time Adaptive Interventions (JITAIs) in Mobile Health: Key Components and Design Principles for Ongoing Health Behavior Support. Ann Behav Med. 2018;52(6):446–62. 10.1007/s12160-016-9830-8. 27663578 PMC5364076

[39] Zhang T, Schoene AM, Ji S, Ananiadou S. Natural language processing applied to mental illness detection: a narrative review. NPJ Digit Med. 2022;5(1):1–13. 10.1038/s41746-022-00589-7. 35396451 PMC8993841

[40] Sobin C, Sackeim HA. Psychomotor symptoms of depression. Am J Psychiatry. 1997;154(1):4–17. 10.1176/ajp.154.1.4. 8988952

