# Supplementary material for: Absolutist word usage in spoken language as a marker of depression: an ecological momentary assessment study
Source: BMC Psychiatry. 2026 Aug 31;26:680. doi: 10.1186/s12888-026-08575-z (PMC13531844; doi:10.1186/s12888-026-08575-z)
Supplement: Supplementary file 1 [file 12888_2026_8575_MOESM1_ESM.docx]

Supplemental Materials for

**Absolutist word usage in spoken language as a marker of depression: an ecological momentary assessment study**

**Figure S1**

Instructions for each ecological momentary assessment (translated)

Hello,

please read the following three questions aloud and respond in whole sentences. Please record both the question and your response.

How are you feeling at the moment?

How are you coping with this feeling?

How would you like to cope with this feeling?

Please provide a rating of your current depressed mood.

0----------10

Please think of a positive thought that has the capacity to improve your mood and record it.

*Note*. Instructions were originally in German.

**Table S1**

List of absolutist words validated in *Al-Mosaiwi et al. (2018)* and word usage frequency

| **Absolutist words** | | **Word usage frequency** | | |
| --- | --- | --- | --- | --- |
| **English (original)** | **German (translation)** | **MDD** | **SC** | **ND** |
| absolutely | absolut(e/er/es/em/en) | 14 | 5 | 14 |
| all, everything | alle(r/s/m/n) | 395 | 416 | 393 |
| always | immer | 263 | 300 | 162 |
| complete | komplett(e/er/es/em/en) | 11 | 17 | 5 |
| completely | vollständig(e/er/es/em/en) | 1 | 4 | 1 |
| constant | konstant(e/er/es/em/en) | 0 | 2 | 0 |
| constantly | ständig(e/er/es/em/en) | 10 | 6 | 0 |
| definitely | definitiv(e/er/es/em/en) | 3 | 3 | 5 |
| entire | gesamt(e/er/es/em/en) | 2 | 3 | 1 |
| ever | jemals | 0 | 0 | 0 |
| every | jede(r/s/m/n) | 71 | 77 | 32 |
| everyone | jedermann | 0 | 0 | 0 |
| full | voll(e/er/es/em/en), völlig(e/er/es/em/en) | 26 | 28 | 23 |
| must | muss/musst/müsst/müssen | 275 | 249 | 191 |
| never | nie, niemals | 7 | 22 | 5 |
| nothing | nichts | 136 | 159 | 139 |
| totally | total(e/er/es/em/en) | 19 | 21 | 5 |
| whole | ganz(e/er/es/em/en) | 884 | 810 | 575 |
| Absolutist word count | | 2,117 | 2,122 | 1,551 |
| Total word count | | 101,483 | 97,618 | 94,743 |

*Note*. When matching participants' utterances to the word list, all relevant German conjugated and declined forms of the absolutist words, shown in brackets, were included. MDD = participants meeting criteria for major depressive disorder; SC = participants with subclinical depressive symptoms; ND = non-depressed participants.

**Table S2**

Formulas applied to calculate absolutist word usage in utterances via Microsoft Excel

| **#** | **Formula** | **Description** |
| --- | --- | --- |
| *Preparing data to count number of words* | | |
| 1 | Removes double spaces | =SUBSTITUTE(*transcription*;” “;” “) |
| 2 | Removes all spaces | =SUBSTITUTE((*transcription*;” “;”“) |
| *Counting words for each transcription* | | |
| 3 | Subtracts number of spaces in #1 from #2 plus 1. | =LEN(*#1*)-LEN(*#2*)+1 |
| *Preparing data to count the number of the respective absolutist word* | | |
| 4 | Adds spaces before and after the transcription | =" "&*transcription*&" " |
| 5 | Counts number of characters in #4 | =LEN*(#4*) |
| *Counting the number of the respective absolutist word* | | |
| 6 | Divides the character count of the absolutist word in the transcription by the character count of the absolutist word. | =(*#5*-LEN(SUBSTITUTE*(#4*;  " "& *absolutist_word*&" ";" "))) /LEN(*absolutist_word*) |
| *Counting the total of absolutist words per utterance* | | |
| 7 | Calculates the sum of all absolutist words in the transcription | =SUM(Σ*#6*) |
| *Calculating the relative absolutist word usage per utterance* | | |
| 8 | Divides total number of absolutist words by total number of words in the transcription | =*#7*/*#3* |

*Note*. Formula #6 was applied for each absolutist word from the list and its conjugations.

**Table S3**

Effects of the relative use of individual absolutist words on depressed mood at t0, t0+1, t0+2, and t0+3

|  | **Model 1 (t0)** | **Model 2 (t0+1)** | **Model 3 (t0+2)** | **Model 4 (t0+3)** |
| --- | --- | --- | --- | --- |
| **Predictor** | ***B*** | ***B*** | ***B*** | ***B*** |
| Depressed mood t0 | -- | 0.43*** | 0.24*** | 0.14*** |
| Negative valence |  |  |  |  |
| Absolut | 41.70*** | -2.29 | 33.11** | 43.02*** |
| Alle | 1.88 | -1.50 | -2.90 | -0.29 |
| Definitiv | -65.07 | 76.77* | 103.00** | 66.72 |
| Ganz | -21.78*** | 2.68 | 5.45** | 3.39 |
| Gesamt | 62.58* | 25.51 | 137.20*** | 278.63*** |
| Immer | -4.23 | -0.26 | 2.68 | 2.47 |
| Jede | -35.29*** | 2.38 | -17.14 | -16.34 |
| Komplett | 54.78** | -52.30** | -118.30*** | -84.33*** |
| Konstant | 308.50 | -281.00 | -226.8 | 231.38 |
| Muss | 3.16 | -2.08 | 4.12 | 1.78 |
| Nichts | 2.97 | -2.46 | 2.57 | 0.37 |
| Nie | -27.22 | 10.95 | 29.48 | 22.18 |
| Ständig | 15.20 | -15.09 | -10.16 | -16.82 |
| Total | 36.44** | -23.59* | -4.96 | -5.87 |
| Voll | -63.17* | -6.50 | 48.22 | 63.76* |
| Völlig | 0.97 | -0.90 | -1.21 | 2.02 |
| Vollständig | -51.92 | 35.27 | 21.06 | 32.90 |
| Positive valence |  |  |  |  |
| Absolut | 19.76 | -17.48 | -2.17 | -12.58 |
| Alle | 1.29 | 0.20 | -0.45 | 0.85 |
| Definitiv | -24.84 | -20.32 | 1.92 | -23.32 |
| Ganz | -4.56*** | 1.16 | 3.70** | 0.55 |
| Gesamt | 0.68 | 15.22 | 2.55 | -26.75 |
| Immer | 3.36 | -0.17 | 0.75 | 2.55 |
| Jede | -0.51 | -2.25 | -0.17 | 1.61 |
| Komplett | 11.72 | -5.45 | 9.25 | -1.01 |
| Konstant | -206.08 | 166.30 | -17.46 | 364.48* |
| Muss | 10.76** | 0.16 | -0.86 | 0.81 |
| Nichts | -3.07 | 3.97 | 4.53 | 2.74 |
| Nie | 6.06 | 15.78 | 9.52 | 9.80 |
| Ständig | 25.99 | 2.56 | -1.26 | -23.79 |
| Total | -10.42 | 1.48 | 3.01 | 2.11 |
| Voll | -11.86 | 9.84 | 17.55 | 5.13 |
| Völlig | -4.91 | 6.74 | 4.03 | 3.04 |
| vollständig | -33.34 | 42.81 | 12.36 | 11.87 |
| Neutral valence |  |  |  |  |
| Absolut | 550.20* | 558.90* | -451.00 | -449.70 |
| Alle | 4.46 | 0.61 | 6.63 | 12.61 |
| Definitiv | 1637.62* | 491.20 | -1509.00 | -859.85 |
| Ganz | -3.65 | -4.63 | 5.65 | 5.55 |
| Gesamt | -61.45 | -451.90* | -129.60 | 82.78 |
| Immer | -3.23 | -3.24 | -4.26 | 8.52 |
| Jede | -37.16 | -16.78 | 3.88 | 11.06 |
| komplett | -47.33 | -41.50 | 418.60 | 176.54 |
| konstant | 5754.04 | -12980.00* | -4165.00 | 1897.90 |
| Muss | -0.69 | -6.71 | -6.56 | 3.65 |
| Nichts | 0.54 | 3.10 | 11.20 | 0.91 |
| Nie | 180.60 | 124.40 | 42.52 | 145.64 |
| Ständig | 67.00 | 27.23 | 80.73 | 84.91 |
| Total | -63.01 | 34.99 | 103.20 | 190.89 |
| Voll | -26.80 | -13.45 | -11.80 | -13.38 |
| Völlig | -179.97 | -232.90 | -81.51 | -345.33 |
| Vollständig | -1807.52 | 959.60 | 932.00 | -37.06 |

*Note*. Effects of individual absolutist words should be interpreted carefully, as some words occurred very rarely (see Table S1).
